# Provision of Genetic Services for Autism and its Impact on Spanish Families

**DOI:** 10.1007/s10803-017-3203-4

**Published:** 2017-07-05

**Authors:** Marta Codina-Solà, Luis A. Pérez-Jurado, Ivon Cuscó, Clara Serra-Juhé

**Affiliations:** 1 Departament de Ciències Experimentals i de la Salut, Universitat Pompeu Fabra, C/Dr. Aiguader, 88, 08003 Barcelona, Spain; 2 Institut Hospital del Mar d’Investigacions Mèdiques, C/Dr. Aiguader, 88, 08003 Barcelona, Spain; 3 Centro de Investigación Biomédica en Red de Enfermedades Raras, Av/Monforte de Lemos, 3-5, Pabellón 11, Planta 0, 28029 Madrid, Spain

**Keywords:** Autism spectrum disorders, Genetic counseling, Genetic services, Genetic diagnosis, Informed decisions, Family planning

## Abstract

Although a genetic evaluation can identify the etiology in 15–30% of individuals with autism spectrum disorder, several studies show an underuse of genetic services by affected families. We have explored the access to genetic services and perception of genetics and recurrence risk in parents of autistic children in Spain. Despite the high interest in genetics, our results show a remarkable underutilization of genetic services, with only 30% of families having visited a genetic service and 13% of patients having undergone the recommended genetic test. This poor service provision influenced recurrence risk perception and had a great impact on family planning. The National Health System should ensure their access to genetic services allowing them to take informed decisions with precise information.

**Electronic supplementary material:**

The online version of this article (doi:10.1007/s10803-017-3203-4) contains supplementary material, which is available to authorized users.

## Introduction

Autism spectrum disorders (ASD) are a group of neurodevelopmental disorders characterized by impairments in three main domains: communication, social interaction and behavior. They are estimated to affect 1 in 68 to 256 children in developed countries, with a male to female ratio of 5:1 (Developmental Disabilities Monitoring Network Surveillance Year 2010 Principal Investigators and Centers for Disease Control and Prevention (CDC) 2014; Taylor et al. 2013). They are among the most inheritable neurodevelopmental disorders, with concordance rates of 60% between monozygotic twin pairs for classic autism, that increase to 90% when considering the broader autistic phenotype (BAP; Bailey et al. 1995; Folstein and Rutter 1977; Lichtenstein et al. 2010; Ritvo et al. 1985; Ronald and Hoekstra 2011; Rosenberg et al. 2009; Steffenburg et al. 1989; Taniai et al. 2008). ASD are considered complex multifactorial disorders, with both genetic and environmental risk factors. Genetic factors involved in ASD comprise all type of genetic variation: chromosomal alterations, triplet expansions, de novo or rare inherited single nucleotide variants (SNVs), and deletions and duplications, known as copy number variants (CNVs). Therefore, ASD present a high degree of genetic heterogeneity, involving various types of genetic variation that might follow several modes of inheritance.

Current guidelines for genetic evaluation of patients with ASD recommend a tiered approach, in which dysmorphology and family history evaluation, in order to identify known syndromes and specific patterns of inheritance, should be the first steps. If no particular condition is suspected, chromosomal microarray (CMA), a high-resolution whole-genome diagnostic tool that detects copy number variants, is indicated in all patients. Fragile X testing should also be considered in males, since this entity accounts for approximately 2% of unselected ASD cases. Overall, a clinical and genetic evaluation including CMA identifies an etiology in approximately 15–30% of individuals (Schaefer and Mendelsohn 2013; Shen et al. 2010). Achieving an accurate genetic diagnosis is of great importance, since genetic counseling based on a specific recurrence risk, as well as reproductive options to minimize the risk on subsequent pregnancies, can be provided to families. In addition, a definite diagnosis can provide information about the prognosis of the disorder and monitor and prevent associated medical conditions. However, in most ASD cases a genetic cause cannot be identified and families must rely on recurrence risk figures based on empirical estimates to take reproductive decisions. When a specific pattern of inheritance cannot be identified, recurrence risk has been estimated to be 18.7–27.7% when considering all the spectrum and 36–50% for families with more than one affected child (Ozonoff et al. 2011; Wood et al. 2015). Due to the complex nature of ASD genetic bases and the relatively low diagnostic yield of current genetic tests, families may experience difficulties understanding and adjusting to information regarding ASD heritability and recurrence risk. Therefore, genetic counseling is relevant both for families in which a genetic cause can and cannot be identified.

Despite the contribution of genetics to ASD, several studies show an underutilization of genetic services by affected families (Selkirk et al. 2009; Shea et al. 2014; Vande Wydeven et al. 2012). This lack of access has a tremendous effect on families, as shown by the consistent overestimation of recurrence risk observed in previous studies (Chen et al. 2014; Mercer et al. 2006; Selkirk et al. 2009; Whitelaw et al. 2007).

Parental misinformation may have a great impact on family planning. In fact, reproductive stoppage—the trend for parents of a child with a severe disability to have none or fewer additional children—has been widely described in ASD families (Grønborg et al. 2015; Hoffmann et al. 2014; Wood et al. 2015). Besides recurrence risk overestimation, underutilization of genetic services could also have an effect on other factors related to family planning, such as perceived causes of ASD and perceived utility of genetic testing. Given the potential consequences of genetic services underutilization, an effort should be made to identify barriers hindering the access of families with ASD to this type of services.

One of such barriers could be the lack of awareness of genetic services, which may vary among countries depending on the professional recognition of such professionals. Therefore, we sought to explore access and perception of genetics in Spain, a country where a public health care system has been implemented, but where genetic professionals are not formally recognized. In 2014, a Royal Decree establishing the professional category and role of clinical and laboratory geneticists was approved, but its implementation has not yet begun. Likewise, genetic counselors are not formally recognized, despite the fact that trained genetic counselors have been practicing for years and a 2-year Master Degree in Genetic Counseling based in Barcelona has been accredited by the European Board of Medical Genetics (Pàmpols et al. 2016; Rantanen et al. 2008). Besides the lack of professional regulation, other factors hindering access to genetic services in Spain may be related to the differences in service provision by the multiple regional administrations of the National Health System and the misinformation and lack of uniform criteria for patient referral among other health professionals.

Therefore, the aim of this research was to explore the access to genetic services and perception of genetics among ASD families in Spain, a country where such studies had not previously been carried out, in comparison with other developed countries.

## Methods and Protocols

### Study Setting and Participants’ Recruitment

The study was addressed to parents with at least one child affected with ASD. Parents were recruited through six ASD family associations in Catalonia, Spain. Board representatives from each association invited its members to participate in an online anonymous survey by email invitation. The survey was accessible between the 6th of August of 2016 and the 1st of October of 2016.

### Instrumentation

The questionnaire was designed taking into account previous studies addressing parents’ knowledge, beliefs and concerns regarding ASD and was reviewed by two genetic counselors, a laboratory geneticist and a clinical geneticist (Supplementary Information 1). Questions addressed different topics: (1) demographics; (2) access to genetics services, previous information and sources of information; (3) perceived causes of ASD; (4) knowledge, opinions and motivation regarding genetics and genetic testing; (5) knowledge and perception of recurrence risk; and (6) impact on reproductive behavior. Types of questions included different formats such as open questions, dichotomous, multiple choice, Likert scales and nominal.

Questions were designed to address topics that would have been covered in a genetic counseling session and that could apply to most genetic disorders. Participants were asked to answer four true/false items, such as “Current genetic tests rule out all causes of ASD” or “A negative genetic test result would mean that recurrence risk in a subsequent pregnancy would be similar to that of the general population” (Supplementary Information 1).

Regarding quantitative risk, parents were asked to estimate their perceived recurrence risk from 0 to 100% in an open question. Qualitative risk perception was evaluated by a forced choice question, in which parents could choose between five different options: null, low, moderate, high or very high risk.

### Data Analysis

Descriptive statistics were calculated for most items. Correlations and statistical significance among selected items were assessed with Chi square and exact Fisher’s tests and t-tests for independent groups. Items of applied knowledge of genetics were rated on a one-point scale (0 points for incorrect or do not know answers and 1 point for correct answers); the total score was the sum of raw scores.

## Results

### Respondents’ Demographics

A total of 130 families agreed to participate in the study and completed the questionnaire (Table 1). Only one of the parents was asked to answer the questionnaire. Therefore, only one answer per family was considered. The survey was mailed to 430 families directly by board members of each Association asking for participation. We obtained a total of 130 complete surveys, with an estimated response rate of 29%.

**Table 1:** Demographic characteristics of our sample

Gender (%)	
Men	14
Women	86
Age (years)	
20–30	6
30–40	33
40–50	50
>50	11
Current relationship status	
Married	87
Divorced	10
Single	2
Widowed	1
Highest level of education obtained	
Primary school	4
High school	32
College graduate	38
Post graduate degree	26
Number of children	
1	42
2	47
3	9
4	2
Diagnosis of affected children	
Autism	55
Asperger	22
PDD-NOS	23

The great majority of families (123/130) had only one affected child, while seven families had two affected siblings. The reported diagnoses of probands were either classic autism (55%, 75/137), Asperger syndrome (22%, 30/137) or pervasive developmental disorder not otherwise specified (PDD-NOS, 23%, 32/137), and their mean age was 7.6 (1–25yo), being 80% (106/137) younger than 10 years old. The mean age of the respondents was 40 years, 86% (112/130) were females, and 80% (104/130) were living with the other parent of the proband. Although the highest level of education obtained varied among participants, 64% (84/130) had completed a college degree. Most of the families had two children (47%). In 42% of the families the affected child was the only child and 11% of respondents had three or more children. Approximately 17% (23/130) of respondents considered that one or more of their relatives were also affected with ASD or a broader phenotype, although most of of them had not been formally diagnosed.

### Access to Genetic Services and Sources of Information

**Fig. 1:**
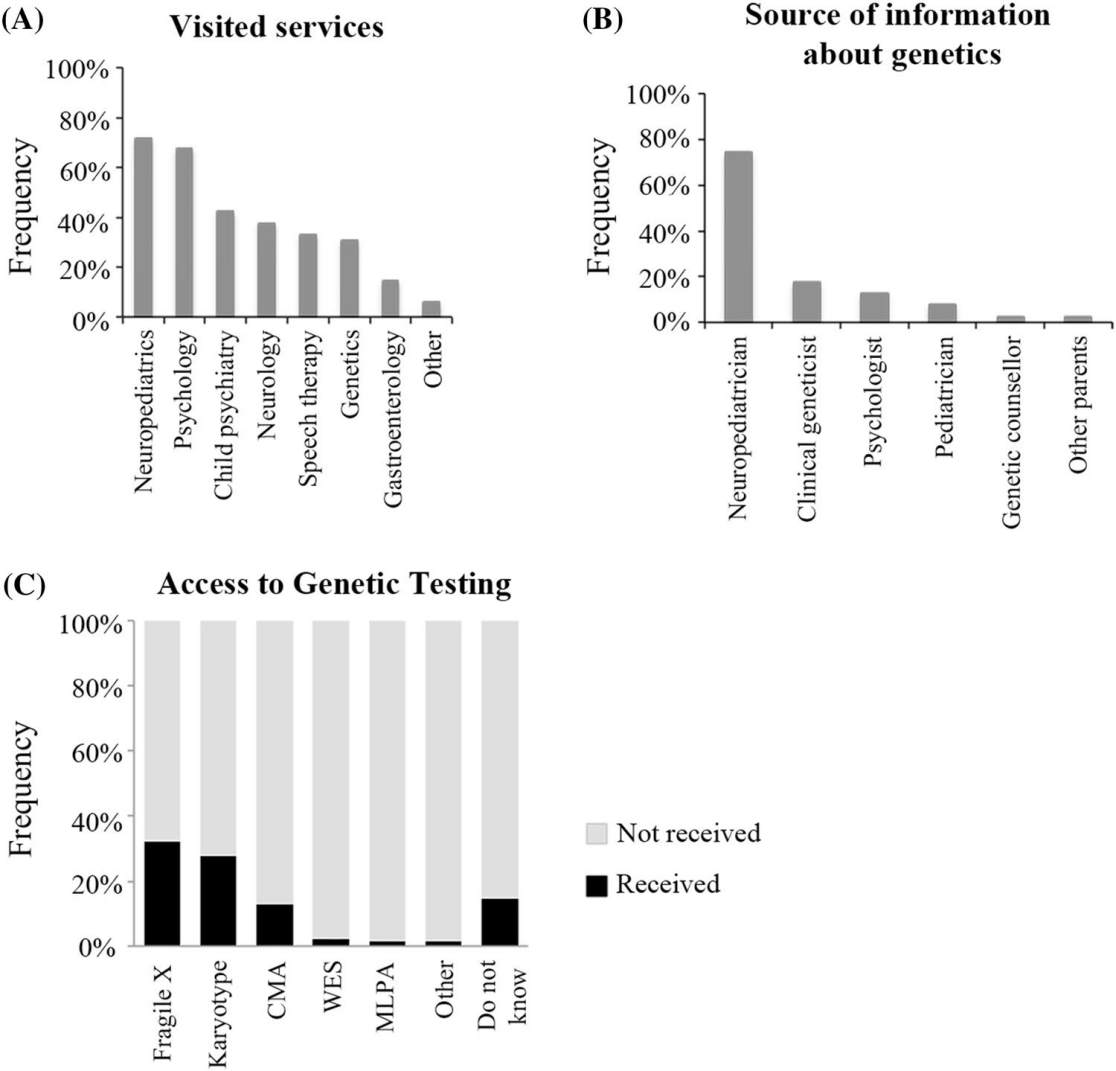
Access to genetic services and information received among families. **a** Most frequently visited services among parents. Less than 40% of parents reported having visited a genetics service. **b** Parents were asked about which professional counseled them about the role of genetics in ASD. The most cited professional was the neuropediatrician. **c** Type of genetic testing received

Only 31% (40/130) of parents reported having visited a genetic service and, surprisingly, only two of the seven families with more than one affected child had been attended by geneticists. The most visited services among parents were neuropediatrics, psychology and psychiatry (Fig. 1a). Moreover, only 22% (28/130) of the participants knew about the professional role of the clinical geneticist and even less (8%, 11/130) about the role of the genetic counselor. Regarding information about the role of genetics and inheritance in ASD, 30% (39/130) of participants stated that they had received some information. Most (74%, 29/39) had been informed by their neuropediatrician, and only 18% (7/39, 5% of the total sample) by a clinical geneticist (Fig. 1b). Finally, only one family had visited a genetic counselor. When asked about the topics they had been informed, 59% of respondents (23/39, 17% of the total sample) reported to be informed about recurrence risk, but only 54% (21/39, 16% of the total sample) about available genetic tests.

Regarding access to genetic testing, 51% (66/130) of participants stated that their child had received some kind of genetic test: fragile X was the most common testing in 64% (42/66), followed by karyotype (36/66) and CMA (17/66), while 29% (19/66) did not know the type of genetic test done (Fig. 1c). Therefore, only 13% of the total sample (17/130) answered their child had received CMA, the first-tier diagnostic tool for the diagnosis of ASD. Not surprisingly, being visited in a genetic service was significantly associated with undergoing genetic testing, since the majority of participants (95%) who had access to genetic services had received genetic testing (${\chi ^2}$, p < 0.0001). It was also interesting that 42% (28/66) of participants who underwent genetic testing had not visited a genetic service. Finally, participants who self-reported their children were diagnosed with Asperger syndrome were less likely to undergo genetic testing, although this figure failed to reach statistical significance (${\chi ^2}$, p = 0.065).

### Perceived Causes of ASD

**Fig. 2:**
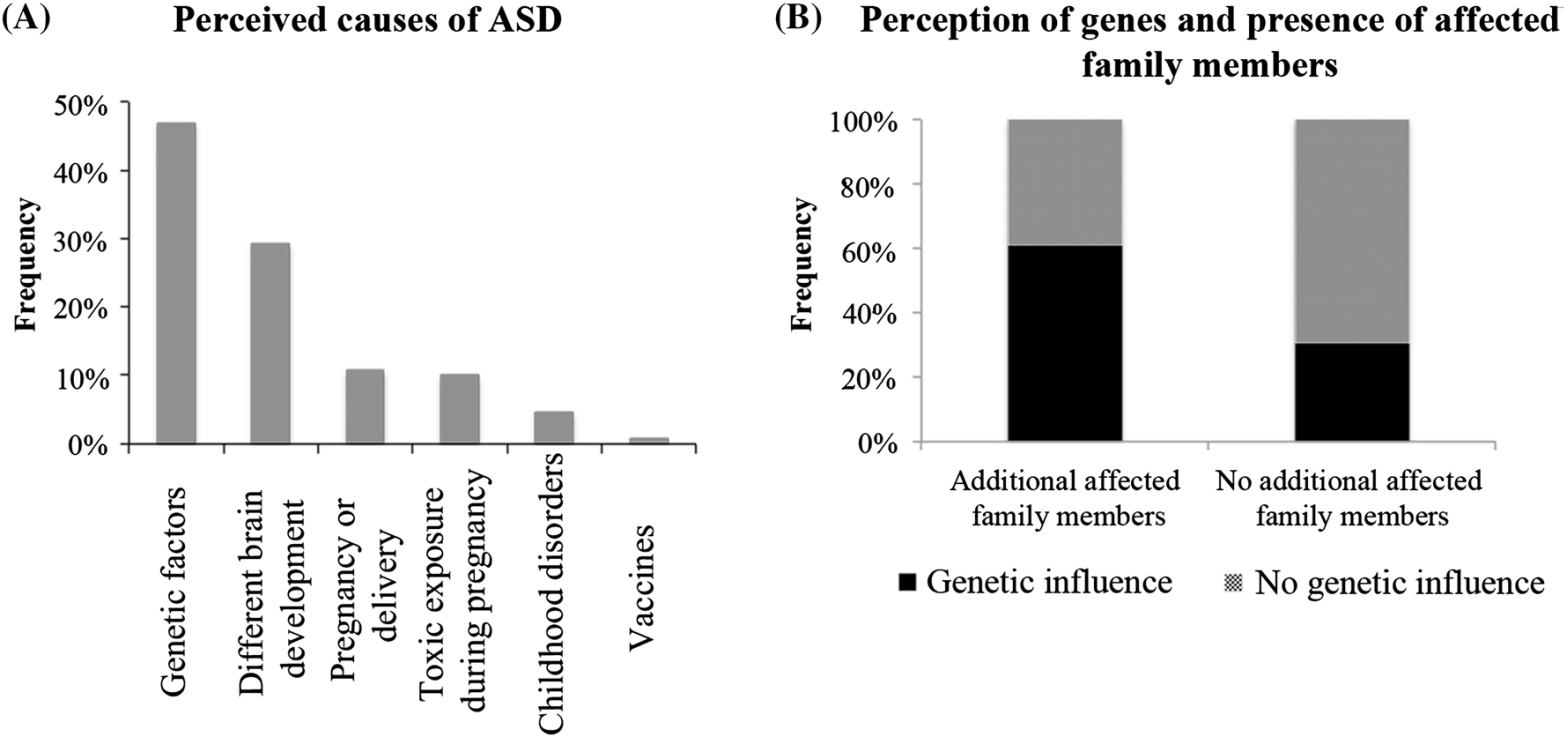
Perception of the influence of genetics in ASD. **a** Perceived causes of ASD among parents. **b** Parents who reported additional family members were more likely to perceive genetics as a possible cause

To evaluate perceived causes of ASD, participants were asked to endorse an unlimited number of items among a list of the most common reported causes of ASD. The most frequent perceived cause was genetic factors (47%, 61/130), followed by different brain development (28%, 4/130), pregnancy or delivery complications (11%, 14/130), exposure to toxic agents during pregnancy (10%, 11/130), early childhood diseases (5%, 6/130) and vaccines (1%, 1/130; Fig. 2a). Perception of genetic factors as causative of ASD was associated with reporting affected relatives (${\chi ^2}$, p = 0.02), since participants self-reporting having an additional affected relative were approximately two times more likely to endorse genetic factors as a cause (Fig. 2b).

### Knowledge, Opinions and Motivations About Genetics and Genetic Testing

Despite only 31% of parents had visited a genetic service, interest in genetics was high, with 95% of respondents (123/130) showing interest in a clinical genetics visit and 94% (122/130) in receiving further genetic testing. Parents were asked to rank their motivations for wanting or not further genetic testing. Among parents who were interested in further genetic testing, the main reasons were: (1) to improve medical management of their affected child (43%, 53/122); (2) to contribute to knowledge and science (39%, 47/122); (3) potential benefits for non-affected relatives (31%, 38/122); (4) to reach a definite diagnosis (27%, 33/122); and (5) to establish the recurrence risk in subsequent pregnancies (22%, 27/122). Motivations for further genetic testing were probably related to social factors, since ranking “establishing the recurrence risk for future children” with a lower punctuation was negatively correlated with age. In fact, participants rating it as most important were, on average, 3 years younger than those who gave it a lower punctuation (Spearman R = −0.4, p = 0.01).

For participants who did not wish further genetic testing (8/130), the main reasons or thoughts were: (1) the disorder could not have a genetic cause (3/8); (2) the results of genetic testing would not improve their child’s well-being (3/8); (3) the cause of the disorder had already been determined (1/8); and (4) they did not want to know whether the disorder was inheritable (1/8).

Concerning questions about genetic knowledge, correct answers exceeded “do not know” and incorrect answers. Total knowledge score correlated with the degree of education (one-way ANOVA, p = 0,004), as well as having been visited in a genetic service (unpaired t-test, p = 0.048). The mean punctuation of parents previously visited in a genetic service was 2.65/4 ± 0.19, compared to 2.17/4 ± 0.17 for those who had not been previously visited.

### Knowledge and Perception of Recurrence Risk and its Impact on Family Planning

**Fig. 3:**
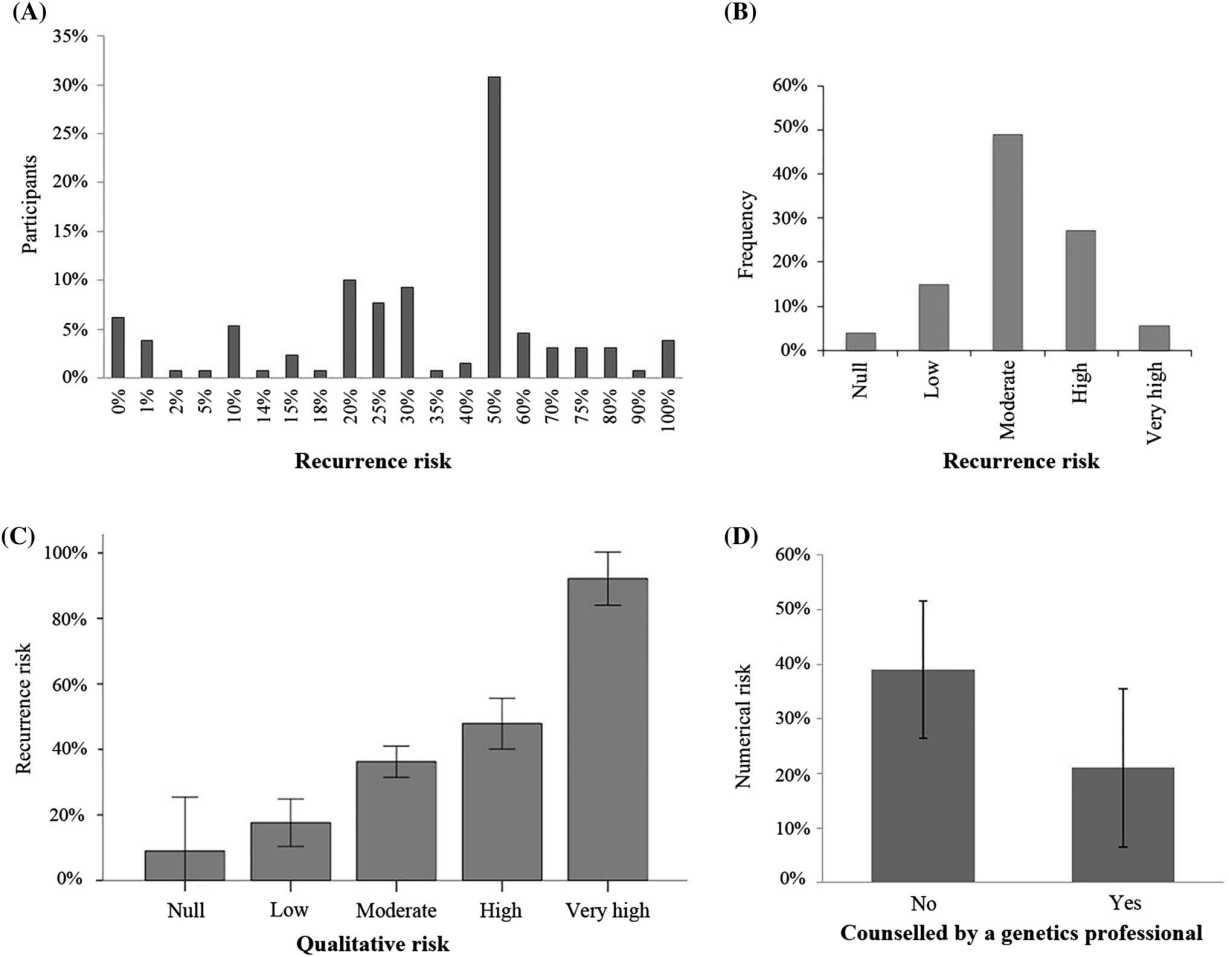
**a** Distribution of perceived risk in numerical terms. **b** Distribution of perceived risk in qualitative terms. **c** Relation between numerical and qualitative risk. Numerical risk is plotted for each qualitative risk category. **d** Numerical risk perception in parents counseled by a genetic professional or by other professionals. Numerical recurrence risk is lower and more accurate among parents counseled by a genetics professional compared to those who were not counseled by a genetic counselor or a clinical geneticist

Perception of recurrence risk was measured in both quantitative and qualitative terms. Participants who reported a known cause were excluded from the analysis; therefore, only families with an unknown diagnosis were included. Regarding quantitative risk estimations, 31% (40/130) of the respondents thought their recurrence risk was exactly 50%, while 18.5% (24/130) answered it was higher than 50% (Fig. 3a). As for qualitative estimates, 49% (63/130) of parents qualified their recurrence risk as moderate (Fig. 3b). Interestingly, quantitative and qualitative risk estimates correlated (Spearman R = 0.57, p = 0.01), although numeric values varied among risk categories (Fig. 3c).

As expected, a higher numerical risk perception was significantly associated with having more than one affected child (unpaired t-test, p < 0.0001), with a mean numerical risk of 36 ± 24% for parents with one affected child and 62 ± 32% for those with two affected children. Respondents with additional family members affected showed a slightly higher risk perception (45 ± 27%) than those who did not (36 ± 25%) was observed, a trend that did not reach statistical significance (unpaired t-test, p = 0.07). Numerical perceived risk was statistically significantly lower among participants counseled by a genetic professional (unpaired t-test, p = 0.043). Participants who had been counseled either by a clinical geneticist or a genetic counselor had a more accurate risk perception (21 ± 29%) compared to those who were not counseled by genetics professionals (39 ± 25%; Fig. 3d).

The majority of participants (62%, 81/130) considered that the risk of having a subsequent child affected had an effect on their reproductive intentions. Of these, 62% (50/81) stated that it had affected “much” their reproductive behavior, 32% (26/81) that it had affected it “quite” and 6% (5/81) that it had “little” effect on their decisions.

**Fig. 4:**
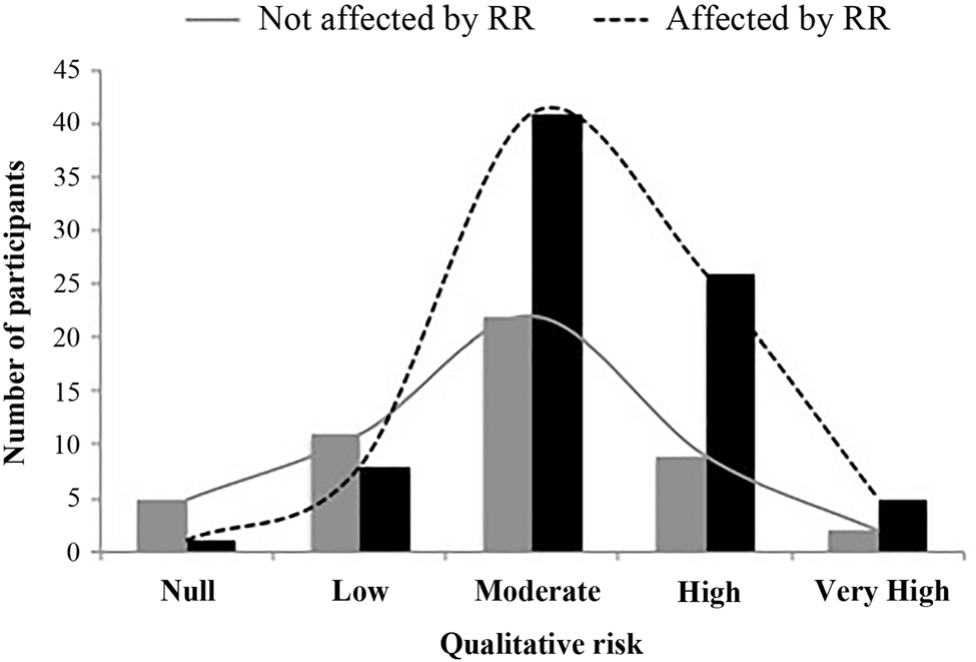
Reported qualitative recurrence risk to have another child with ASD (RR) is compared between parents affected (*black*) and not affected (*grey*) by recurrence risk. Qualitative recurrence risk perception was higher among parents who reported an effect on family planning

We also asked parents for reasons influencing their reproductive behavior (Table 2). Among those who believed their recurrence risk was high, the main reason affecting their family planning was “fear of having a subsequent affected child” (72%, 59/81). Statistical analyses were carried out to assess if higher risk perception was associated to self-reported effect on reproductive behavior. Parents who answered that perception of recurrence risk had an effect on family planning had a higher risk perception (42 ± 26%) than those who did not (32 ± 25%), both in numerical (unpaired t-test, p = 0.045) and qualitative terms (${\chi }^{2}$, p = 0.024) (Fig. 4).

**Table 2:** Reasons influencing reproductive behavior among parents affected by recurrence risk and not affected

	Effect on FP (%)	No Effect on FP (%)
Fear of having another affected child	73	4
Time devoted to a new child	58	8
Economical resources devoted to a new child	51	12
Efforts devoted to a new child	57	8
Completed family	5	20
Having an affected child has not influenced my reproductive decisions	5	43
Infertility	0	6
Did not know diagnosis at the time	1	6
Age	0	4
Other	5	8

*FP* Family planning

## Discussion

According to data gathered from 130 parents with a child with ASD living in Catalonia, Spain, genetic services are clearly underutilized. Our data are similar to those previously reported in other Western countries. (Mercer et al. 2006; Selkirk et al. 2009; Vande Wydeven et al. 2012). A study carried out in USA in 2009 reported that only 24% of parents whose child was diagnosed with ASD had seen a genetic counselor (Selkirk et al. 2009). More recent studies based also in USA reported similar rates of utilization of genetic services by families of ASD patients, ranging from 20–28% (Amiet et al. 2014; Chen et al. 2013; Cuccaro et al. 2014; Vande Wydeven et al. 2012). It was hypothesized that the low utilization rate was mostly due to a lack of referral, possibly related to an extended unawareness of the clinical utility of genetics for ASD among other professionals (Salm et al. 2014). To our knowledge, only one study assessing utilization of genetic services has been carried out in Europe, specifically in France, reporting a utilization rate of 60%, significantly higher than in USA and Spain (Amiet et al. 2014). This disparity might be partly explained by the differences in health care systems between USA and France (Amiet et al. 2014). However, in Spain like in France, there universal health coverage by the Public Health Service including genetic testing if required. Therefore, additional factors such as the disparity among countries in the regulation of genetic professions and the different awareness among other professionals involved in the care of ASD patients might explain the difference. In France, genetics was recognized as a medical specialty in 1995 and the Law of Public Health legally recognizes genetic counselors as health professionals since 2004, whereas such formal regulation does not exist in Spain. This lack of regulation could also have an impact on the awareness of such services of other professionals and the general population, including affected families. In fact, only 22% of the respondents in our study knew about the professional role of the clinical geneticist and an even lower figure (8%) about genetic counselors.

The underutilization of genetic services may result in several potentially harmful consequences for families. First, several identifiable genetic causes may be missed by the current approach. It is estimated that a genetic diagnosis may be reached in 30% of cases when patients undergo a clinical evaluation and the first tier test, CMA. In our cohort, only 30% of patients had been visited in a genetics service and barely 13% underwent CMA. Consequently, several families with an identifiable genetic alteration may have been missed and could not benefit from specific recurrence risks and associated reproductive options to minimize their risk in future pregnancies.

Second, in our study a large fraction of genetic tests was requested outside a genetic service and probably without appropriate prior genetic counseling. This has also been observed in a study carried out in USA, which showed that genetic testing was often ordered in the absence of the accompanying genetic services (Cuccaro et al. 2014). Genetic tests that scan the whole genome, such as CMA or exome analysis, involve a significant amount of complexity and may identify incidental findings, variants of unknown significance and variants of variable expression and incomplete penetrance. The complex genetic landscape of ASD makes these scenarios even more difficult to manage and require multidisciplinary teams with expertise in the field. Moreover, with the advent of whole exome sequencing, genetic testing options are likely to increase and so will do its associated complexity. Consequently, a correct interpretation of the results along with the proper communication to families will become even more important. If such results are not properly communicated, they may result in parents misunderstanding of the role of genetics in ASD.

Third, inaccurate information regarding the influence of genetics in ASD may also have an effect in family planning. Previous studies have shown that reproductive stoppage, the trend for parents to have fewer children after the diagnosis of a condition, is frequent in families with children with ASD (Grønborg et al. 2015; Hoffmann et al. 2014; Wood et al. 2015). In fact, 62% of the parents of our study admitted that the risk of recurrence had an effect on their family planning. When asked about what reasons were influencing family planning, the main reason for parents who felt recurrence risk had an effect on family planning was “fear of having an affected child”. This is in consonance with other studies showing that a great majority (90%) of individuals who wanted more children felt that recurrence risk had significantly affected their decision for family planning (Selkirk et al. 2009). Participants in our study also had a highly inaccurate risk perception, since most parents estimated their recurrence risk at 50% (Chen et al. 2014; Selkirk et al. 2009). Moreover, parents who felt their risk had affected their reproductive decisions, tended to perceive it as high than those who did not, suggesting that for parents with lower risk perception, recurrence risk does not play such an important part in family planning decisions. Of course, recurrence risk perception is not the sole factor influencing family planning, since parents caring of a child with ASD also experience higher levels of stress, even compared to those with children with other developmental needs (Keenan et al. 2016; Schieve et al. 2007). Anyway, our data suggests that recurrence risk perception plays an important role in parents’ decisions.

Accordingly, parents should clearly benefit from discussing recurrence risk with trained professionals such as genetic counselors and/or clinical geneticists, given that their reproductive decisions may change after receiving appropriate risk figures. Indeed, parents who had been counseled by either a clinical geneticist or a genetic counselor had more accurate risk perception, lower than those who were not counseled. Moreover, significant differences in knowledge were also observed between families who had or had not visited a genetic service. Those who had visited a genetic service obtained a higher mean punctuation (0.5 points on a total scale of 4), showing that they had received more comprehensive information. Therefore, genetic testing and counseling may be useful for parents, especially if offered at the right time. In order to explore whether the low access to genetic services previously discussed was due to parental disinterest or to other factors such as lack of referral, we asked parents about their interest in being seen by a genetics provider. An overwhelming majority (95%) answered favorably, including participants who had already been visited, and half of parents in our study (47%) believed that genetic factors were one of the main causes of ASD, similar to previous studies conducted in Western countries (Mercer et al. 2006; Selkirk et al. 2009).

This is the first study evaluating genetics perception among parents of children affected with ASD conducted in Spain. Since participants were recruited through parental associations and participation was voluntary, our sample may be biased, although most likely towards more informed families. In addition, although we excluded families with known causes from recurrence risk perception analysis, our figures could be slightly overestimated, since some of the participants may have refused to share their genetic test results. Despite these limitations, we believe this work adds to the body of research in a country where no similar studies had been conducted before, and significant efforts have still to be made to translate genetic research into clinical practice.

In conclusion, the results of this study show a clear underutilization of genetic services among families of children with ASD in Spain, despite their widespread view that genetics is one of the major causative factors and their interest in genetic testing. Most patients did not have access to the recommended tiered diagnostic approach that may reach a diagnosis in up to one-third of cases, a fraction that is expected to increase with the widespread clinical implementation of exome and genome sequencing. This lack of service provision may have some negative effects on clinical follow-up and more clearly on family planning. An important fraction of patients and families would benefit from achieving a molecular diagnosis and, if not reached, all would benefit from appropriate genetic counseling with empiric information about recurrence risk. Underutilization of genetic services has also been reported in other Western countries, such as the USA and to a less degree in France (Amiet et al. 2014). It is a responsibility for Health Authorities and the genetics community to ensure that all families with ASD can have access genetic services. Measures to improve the awareness of the role and relevance of genetics in ASD should addressed to both the general population and other health professionals involved in the care of these patients, which might contribute to increase referral rates to genetic services. In addition, other measures are required to minimize disparities between countries. In Spain, there is also still a need for the establishment of formal training programs and the legal regulation and recognition of the genetic professions.

## Electronic supplementary material

Below is the link to the electronic supplementary material.

Supplementary material 1 (DOCX 25 KB)
