# Supplementary material for: Provision of Genetic Services for Autism and its Impact on Spanish Families
Source: J Autism Dev Disord. 2017 Jul 5;47(10):2947–56. doi: 10.1007/s10803-017-3203-4 (PMC5602032; doi:10.1007/s10803-017-3203-4)
Supplement: Supplementary file 1 — Supplementary material 1 (DOCX 25 KB) [file 10803_2017_3203_MOESM1_ESM.docx]

**Supplementary Information**

**Provision of genetic services in ASD Spanish families**

The aim of this study is to better understand previous knowledge, opinions and needs regarding genetics of families with a child with autism spectrum disorder (ASD). The data obtained through this study will be treated completely anonymously. The final purpose of the study is to improve access to genetic units and healthcare.

**1. In the course of your child’s disorders, which hospital services have you visited at least once?**

Child psychiatry

Psychology

Neurology

Neuropediatrics

Gastrodigestive services

Genetics

Logopedics or phoniatrics

Others (please specify): _________

**2. Have you received previous information about the role of genetics in ASD?**

Yes

No

**3. Which professional has counseled you about this topic?**

My general practitioner

A pediatrician

A neurologist

A clinical geneticist

A genetic counselor

Others (please specify): ________

**4. Which topics have you received information about?**

Recurrence risk in a subsequent pregnancy

Available genetic tests for its molecular diagnosis

Cause of the disorder

About the diagnosis and the natural course of the disorder

**5. Do you know about the professional role of the clinical geneticist?**

Yes

No

**6. Do you know about the professional role of the genetic counsellor?**

Yes

No

**7. Would you be interested in being visited in a genetic services and receive information about the role of genetics in ASD?**

Yes

No

**8. Are you part of a family or patients’ ASD association?**

Yes

No

**9. Which is the name of the association which you belong to?**

Please specify: _________

**10. Which of these factors do you believe can cause Autism Spectrum Disorders?**

Exposure to toxic factors during pregnancy

Genetic factors

Medical complications during pregnancy or delivery

Alternative brain development

Vaccines

Other childhood diseases

Other (please specify):_______

**11. Has your child undergone some kind of genetic testing?**

Yes

No

**12. Which kind of genetic testing has your children undergone?**

Fragile X

Karyotype

array CGH

Exome sequencing

MLPA

Others (please specify): ________

I do not know

**13. With regard to the implications of a genetic testing, which of these statements do you believe are true or false**?

Genetic tests can detect all causes of Autism Spectrum Disorders.

If all tests have obtained negative results, the chance of having an affected child in a subsequent pregnancy is very low, similar to that of the general population.

Prenatal diagnosis is possible in a subsequent pregnancy, even if the genetic cause has not been identified in the first affected child.

The result of a genetic test can have implications for other family members.

**14. Would you like to receive further genetic testing that could determine the cause of your child’s disorder?**

Yes

No

**15. What are your motivations for pursuing further genetic testing? Please rate them for 1 to 5, where 5 is the most important and 1 is the least important.**

a) Obtain a definite diagnosis

b) Improve the treatment and medical management of my affected child.

c) Establishing the recurrence risk for a subsequent pregnancy and learn about reproductive options to avoid having an affected child

d) Benefits for other family members, such as establishing their own recurrence risk

e) Contributing to knowledge and science

**16. What are your motivations for not wanting further genetic testing?**

I believe that the results would not improve my child’s treatment.

I do not wish to known if the cause of the disorders is inheritable.

I do not believe my child’s disorder has a genetic cause

My child’s disorder cause has already been determined

**17. From 0% to 100%, what do you think would be your probability of having a subsequent affected child?**

Risk: [open question]

**18. In numeric terms, what would be the risk of having another affected children?**

Null risk

Low risk

Moderate risk

High risk

Very high risk

**19. In case you would have a subsequent child, what would be his or her degree of affectation?**

I do not know

It is not possible to know

Higher

Lower

Similar

**20. Do you think that the risk of having an affected child has influenced your family planning?**

Yes

No

**18. How much has this risk affected your family planning?**

A little

Somewhat

Much

**19. What reasons have influenced your family planning?**

Fear of having another affected children

Having another child would prevent me from spending time to my affected child

Having another child would prevent me from devoting economical resources to my affected child

Having another child would prevent me from devoting enough effort to my affected child.

Having an affected child has not influenced my reproductive behavior

I did not wish to have further children, whatever their degree of affectation

Other (please specify):____________

**Demographic information:**

**1. Age:______**

**2. Gender:**

Male

Female

**3. Which is your higher degree of education?**

Primary education

Secondary education

College education

Postgraduate degree

**4. Which is your current civil status?**

Married or living with a partner

Single

Widowed

Divorced

Other (please specify): _________

**5. How many children do you have?**

One

Two

Three

Four

More than four

**6. Which are the age, gender and diagnosis of your children?**

| **Children** | **Age** | **Gender** | **Diagnosis** |
| --- | --- | --- | --- |
| Older child | Open question | Male/Female | Not affected/classic autism/Asperger/PDD-NOS |
| Second oldest child |  |  |  |
| Third oldest child |  |  |  |
| Fourth oldest child |  |  |  |

**7. Do you have additional affected family members?**

a) Yes

b) No

If yes, what is your familial relationship with them? [open question]
